# Phosphorylation of the Retinoic Acid Receptor Alpha Induces a Mechanical Allosteric Regulation and Changes in Internal Dynamics

**DOI:** 10.1371/journal.pcbi.1003012

**Published:** 2013-04-18

**Authors:** Yassmine Chebaro, Ismail Amal, Natacha Rochel, Cécile Rochette-Egly, Roland H. Stote, Annick Dejaegere

**Affiliations:** 1 Institute of Genetics and Molecular and Cellular Biology, Integrated Structural Biology Department, Illkirch, France; 2 Institute of Genetics and Molecular and Cellular Biology, Functional Genomics and Cancer Department, Illkirch, France; Mount Sinai School of Medicine, United States of America

## Abstract

Nuclear receptor proteins constitute a superfamily of proteins that function as ligand dependent transcription factors. They are implicated in the transcriptional cascades underlying many physiological phenomena, such as embryogenesis, cell growth and differentiation, and apoptosis, making them one of the major signal transduction paradigms in metazoans. Regulation of these receptors occurs through the binding of hormones, and in the case of the retinoic acid receptor (RAR), through the binding of retinoic acid (RA). In addition to this canonical scenario of RAR activity, recent discoveries have shown that RAR regulation also occurs as a result of phosphorylation. In fact, RA induces non-genomic effects, such as the activation of kinase signaling pathways, resulting in the phosphorylation of several targets including RARs themselves. In the case of RARα, phosphorylation of Ser369 located in loop L9–10 of the ligand-binding domain leads to an increase in the affinity for the protein cyclin H, which is part of the Cdk-activating kinase complex of the general transcription factor TFIIH. The cyclin H binding site in RARα is situated more than 40 Å from the phosphorylated serine. Using molecular dynamics simulations of the unphosphorylated and phosphorylated forms of the receptor RARα, we analyzed the structural implications of receptor phosphorylation, which led to the identification of a structural mechanism for the allosteric coupling between the two remote sites of interest. The results show that phosphorylation leads to a reorganization of a local salt bridge network, which induces changes in helix extension and orientation that affects the cyclin H binding site. This results in changes in conformation and flexibility of the latter. The high conservation of the residues implicated in this signal transduction suggests a mechanism that could be applied to other nuclear receptor proteins.

## Author Summary

Allosteric regulation of proteins is critically important in many biological processes. Here we focused on the allosteric pathway of communication within a ligand-regulated transcription factor, the Retinoic Acid Receptor (RAR). Recent experimental studies performed with the RARα subtype have shown that phosphorylation of a residue located at one extremity of an α-helix in RAR, leads to a changes in binding affinity at the other extremity of the same helix for cyclin H, a binding partner that is necessary for gene transcription activation. The purpose of our study was to understand the conformational changes occurring within the receptor upon phosphorylation. Molecular dynamics simulations are well suited for this sort of study. Through this approach, we were able to show that although the overall structure of the phosphorylated RAR shows no distinct difference from the unphosphorylated form, evidence is provided for an allosteric regulation pathway that implicates more subtle changes, such as changes in side chain orientations, which affect the internal dynamics of the receptor.

## Introduction

**Figure 1:**
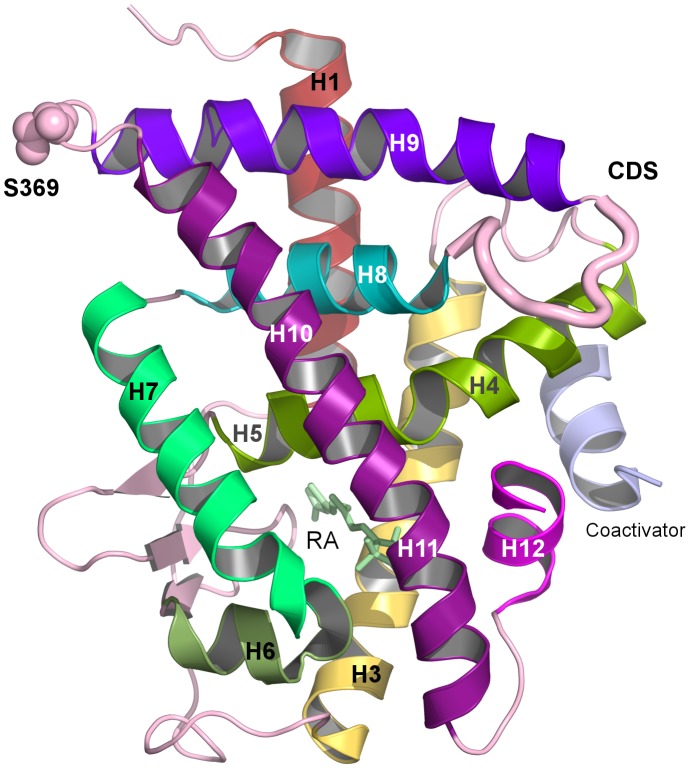
Structural representation of the ligand-binding domain of RARα illustrating the cyclin H docking site (CDS) and the phosphorylation site (S369).

Nuclear receptors are ligand-dependent transcription factors that participate in many cellular signaling networks involved in various physiological phenomena, such as embryogenesis, cell differentiation, cell growth, reproduction and apoptosis [1]. Disruption or abrogation of these signaling pathways results in a variety of diseases or in malignant cell transformation. The superfamily of nuclear receptors includes the nuclear retinoic acid receptors (RARs), which bind retinoic acid (RA), the active metabolite of vitamin A and which function as heterodimers with a second family of nuclear receptors, the retinoid X receptors (RXRs). There are three RAR and RXR subtypes (RAR-α, -β, -γ and RXR-α, -β, -γ) [2] and they regulate gene expression by binding as RXR/RAR heterodimers to retinoic acid response elements (RAREs) located in the promoter regions of target genes [3]–[5]. RARs and RXRs display a well-defined domain organization, composed mainly of an unstructured N-terminal domain (NTD) and two well-structured domains, a central DNA-binding domain (DBD) and a C-terminal ligand-binding domain (LBD). The DBD is composed of two zinc finger motifs and two α-helices. The LBD is formed by 12 α-helices and one β-sheet, which display the general three layer antiparallel helical sandwich fold found in the NR superfamily (see Figure 1).

The general scenario of RAR activation starts with the binding of the ligand to the LBD and the subsequent departure of co-repressor and recruitment of co-activator proteins [3]. Crystallographic structures and the characterization of co-regulatory complexes, such as Topoisomerase II [6] for repression and DNA-dependent ATPases for activation processes [7] have provided a wealth of information on how RARs regulate transcription. Multiple structures of individual DBDs and LBDs have been determined [8]–[12]. Recent X-ray structures [11], solution state structures by SAXS [13] and cryo-EM structures [14] of full-length nuclear receptor protein complexes continue to increase our understanding. The binding of agonist ligands, such as retinoic acid (RA), to the LBD induces large-scale conformational changes, the most prominent being the repositioning of the C-terminal helix H12. This particular structural rearrangement results in the exposure of a binding site for co-activator proteins that contain the LXXLL consensus motif [15]. Allosteric communication pathways have been identified *in silico* between the ligand and the co-activator peptide [16] and between functionally relevant protein interfaces [17]. This fine-tuned regulation process leads to the recruitment of several protein complexes with enzymatic activities, such as histone actelytransferases and DNA-dependent ATPases [18], which subsequently induce alterations in the chromatin structure around the target genes promoters.

Besides this classical mode of genomic effects, RA also has non-genomic and non-transcriptional effects exemplified by the activation of p38MAPK/MSK1 pathway [19]–[21]. Activation of this pathway results in the phosphorylation of the RARα ligand-binding domain at serine 369 (S369), located in loop L9–10 within the LBD [22]. This post-translational modification leads to an increase in the binding affinity of the LBD domain for cyclin H [23]. Cyclin H, together with cdk7 and MAT1, form the Cdk-activating kinase (CAK) subcomplex of the general transcription factor TFIIH, which is involved in transcription initiation and DNA repair. Binding of cyclin H to the RARα LBD positions the CAK complex so that the cdk7 kinase can phosphorylate a second serine of RARα (S77) located in the NTD [24], [25]. The phosphorylation of this N-terminal residue is required for the recruitment of RARα to target genes promoters [21].

In a recent study, we showed that phosphorylation of S369 leads to changes in the structural dynamics of the cyclin H binding site, composed of the loop between helices 8 and 9. This change in dynamics was correlated to the increase in the cyclin H/RARα binding affinity [26]. Given that S369 is located almost 40 Å from the binding site of cyclin H, an allosteric mechanism clearly makes an important contribution to this overall process. Interestingly, S369 is essentially absent outside of mammalian RARα indicating that this fine-tuned phosphorylation cascade appears late during vertebrate evolution [25].

In this work, we use molecular dynamics simulations to elucidate the effects of phosphorylation on the conformational flexibility and dynamics of the RARα LBD and to identify the factors that compose the allosteric signal. An extensive analysis of the effects of phosphorylation on atomic fluctuations, salt bridges and ion-pair networks was coupled to quasi-harmonic and correlated motions analyses. The aim was to identify the consequences of phosphorylation on the structural dynamics. From this analysis, we proposed a coherent and complete picture of the allosteric communication between the S369, the phosphorylation site, and the cyclin H binding site.

## Results

### Phosphorylation of Ser369 does not induce overall major structural modifications in the LBD of RARα

As a measure of the structural stability of RARα, the overall root mean square deviation (RMSD) of the backbone coordinates was calculated as a function of time and averaged over the three simulations of the phosphorylated and non-phosphorylated protein, respectively. This measure was done relative to the initial energy-minimized structure, which was the same in all simulations. The time series were calculated over the 50 ns of dynamics (see Figure S1) and showed characteristic behavior of stable simulations with no overall drift. Mean values of 1.13 Å and 1.14 Å for phosphorylated RARα (p-RARα,) and unphosphorylated RARα (unp-RARα), respectively, were calculated, suggesting that phosphorylation does not induce major structural changes implicating backbone reorganization.

This stable behavior is coherent with the cluster analysis of the trajectories [27], [28] that was performed in order to evaluate whether the trajectories of the phosphorylated and unphosphorylated forms converge to a representative ensemble of structures. The detailed description is given in Material and Methods. Indeed, the cluster analysis indicated good convergence of the structural ensembles on the timescale of the simulations.

Finally, the local structural deviations were also analyzed from average structures extracted from the last 10 ns of the simulations. The local backbone RMSD from the initial structure was calculated on a per-residue basis (see Figure S2). No major differences were observed between the phosphorylated and unphosphorylated forms, except in the vicinity of S369 (i.e. loop L9–10) where the RMSD is smaller in the case of the phosphorylated protein. This suggests that phosphorylation stabilizes to some extent loop L9–10.

Together, these results suggest that phosphorylation of S369 does not lead to any significant changes in the overall conformation of RARα whereas only small, localized changes in the receptor's backbone conformation occur in the loop L9–10.

### Ser369 phosphorylation modulates a local salt bridge network

Salt bridges play an important role in nuclear receptor structure. A recent structure-based sequence analysis revealed differentially conserved salt-bridges that partition the NR superfamily into two classes related to their oligomeric behavior [29]. Heterodimer-forming receptors, such as RARα belong to class II where, following the nomenclature in [29], conserved salt bridges are formed between, i) E/D42 in H5 and R62 in loop L8–9 and, ii) E50 in H8 and R/K/H90 in H9. Interestingly, these conserved salt bridges involve residues situated in the vicinity of the phosphorylation site (loop L9–10) and of the cyclin H docking site (loop L8–9) [23], [29], suggesting their possible involvement in the allosteric mechanism between the two sites.

**Figure 2:**
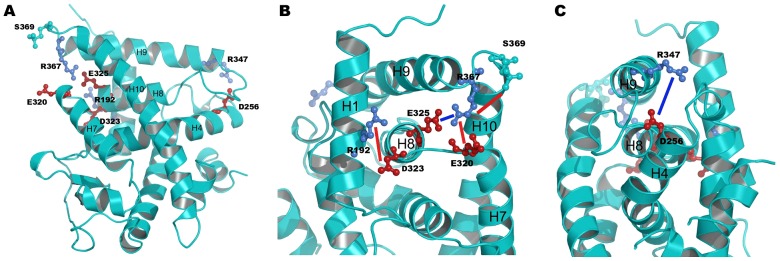
Positions of the identified residues implicated in the salt bridge and hydrogen bond network (R192, D256, E320, D323, E325, R347, R367, S369). The residue pair R347-D256 is located around the cyclin H binding site in loop L8–9 and the residue pairs S369-R367, E325-R367, D232-R192 and R320-R367 are located in the vicinity of the phosphorylation site in loop L9–10 (A). Blue lines illustrate the distances that decrease upon phosphorylation and red lines illustrate the ones that increase around the phosphorylation site (B) and around the cyclin H binding site (C).

**Figure 3:**
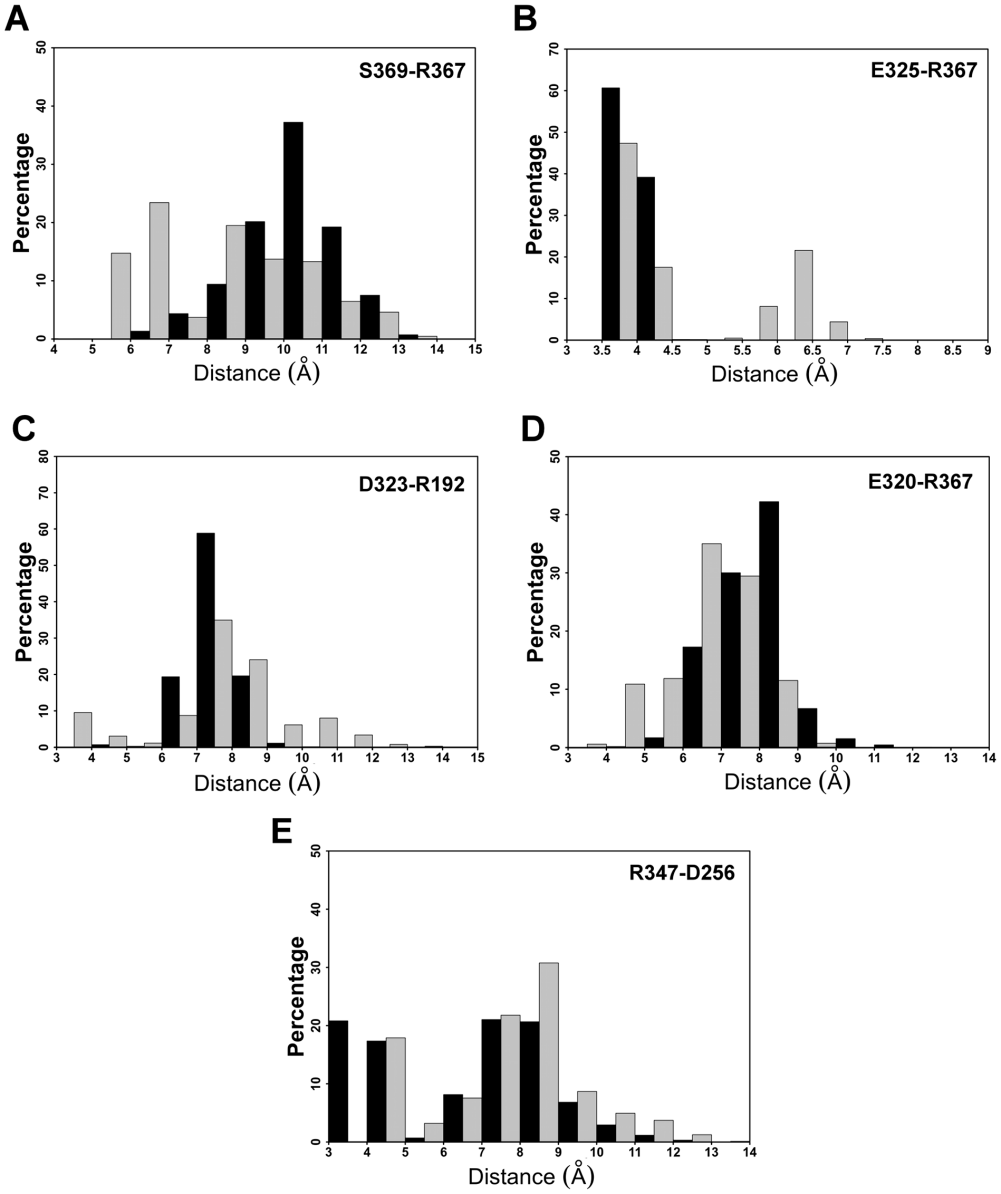
Histograms of the following four distances: S369-R367 (A), E325-R367 (B), D323-R192 (C), E320-R367 (D), and R347-D256 (E), for the unphosphorylated RARα (in black) and the phosphorylated RARα (in grey).

Here we analyzed the impact of S369 phosphorylation on the formation of salt bridges networks in the RARα LBD. The analysis was performed by monitoring the distances between carbon atoms of partner residues within a salt bridge (Cζ atom of Arg, Cε of Lys, Cδ and Cγ for the Glu and Asp, respectively). We monitored all salt bridge/ion pairs of the receptor LBD and found that five out of a total of 35 were affected upon S369 phosphorylation (see Figure 2 for a representation of the amino acids involved and Figure 3 for the associated structural changes upon phosphorylation).

A first group of affected interactions is situated in the vicinity of the phosphorylation site. Phosphorylation of S369 introduces a negative local charge that is felt by the nearby R367 (see Figure 2.A for the structure) and as a result, conformations with shorter distances between the Oγ of S369 and Cζ of R367 are observed in the phosphorylated receptor as compared to the non-phosphorylated (see Figure 3.A). This reorientation of R367 affects its interactions with nearby acidic amino acids. The hetero-dimer specific [29] salt bridge E325–R367 (E42–R62 in the unified nomenclature above, see Figure 2.A) is not systematically formed in the phosphorylated receptor, indeed the salt bridge separation distances that fall between 3.5 and 4.5 Å account for 65% of the population in the p-RARα simulations, as opposed to 99.8% in the non-phosphorylated form (see Figure 3.B). In addition, a new maximum located between 6 and 6.5 Å with a population of 22% is observed in p-RARα (see Figure 3.B).

The disruption of the class-specific salt bridge is accompanied by the formation of a new ionic interaction between R367 and E320, which is situated close to E325, but oriented towards the surface of the receptor LBD (see Figures 2.A, 2.B and 3.D). This is reflected in the dominant distance peak moving from 8–9 Å (42% of the conformations) in the unp-RARα simulations to 6–7 Å (35% of the conformations) in the p-RARα simulations (see Figure 3.d). In addition, a new minimum separation distance between 4–5 Å appears with p-RARα (11%, see Figure 3.D). Another affected salt bridge in the phosphorylation region concerns E323 and R192. E323 is situated in H8 while R192 is in H1, in the N-terminal region of the receptor (see Figure 2.B). Phosphorylation leads to a decrease of the separation distance between these two residues. In comparison to the distance distribution in the unphosphorylated form, a new peak containing 9.5% of the conformations appears between 3 to 4 Å, whereas the dominant peak between 7 to 8 Å decreases from 59% in the unp-RARα form to 35% in p-RARα (see Figure 3.C). Overall, distances that are above 7 Å account for 80% of the conformations in unp-RARα and 77% in p-RARα.

Besides these four perturbed ionic interactions in the vicinity of the phosphorylation site, an ionic interaction formed between D256 in the N-terminal part of H4 and R347 in the N-terminal region of H9 (see Figures 2.A and 2.C) is perturbed upon phosphorylation as well. In the unphosphorylated simulations, the salt bridge D256–R347 is formed in 21% of the conformations with short (3 to 4 Å) D256-Cγ to R347-Cζ distances (see Figure 3.E). Upon phosphorylation, the D256-Cγ to R347-Cζ distances increase to values above 4 Å (see Figure 3.E). The separation distances larger than 5 Å represent 82% of all values in the phosphorylated form of RARα, as opposed to 62% in the non-phosphorylated form of RARα (see Figure 3.E). These results suggest that phosphorylation of S369 induces weakening of the R347–D256 salt bridge. In addition, when the distance between D256 and R347 increases, the donor-H-acceptor angle between the two N atoms of R347 and the two O of D256 is disrupted and becomes lower than 120°, further supporting the breaking of the salt bridge in the phosphorylated simulations (data not shown). An important point is that this latter salt bridge is situated in the region of the cyclin H binding site, about 40 Å from the phosphorylated serine.

Together, the modified ionic-pair patterns show that the phosphorylation has a tendency to remodel the network of salt bridge and ion-pair interactions near the phosphorylation site. These changes result in an approaching of the C-terminal end of H8 to H1, as well as of L9–10 to the C-terminal part of H7. By contrast, phosphorylation leads to changes around the cyclin H binding site, where salt bridge distances increase between the N-terminal part of H4 and the N-terminal part of H9, suggesting a loosening of the structure in this region. These changes are illustrated in Figures 2.B and C where the blue lines represent distances that increase and red lines represent distances that decrease upon phosphorylation of RARα.

**Figure 4:**
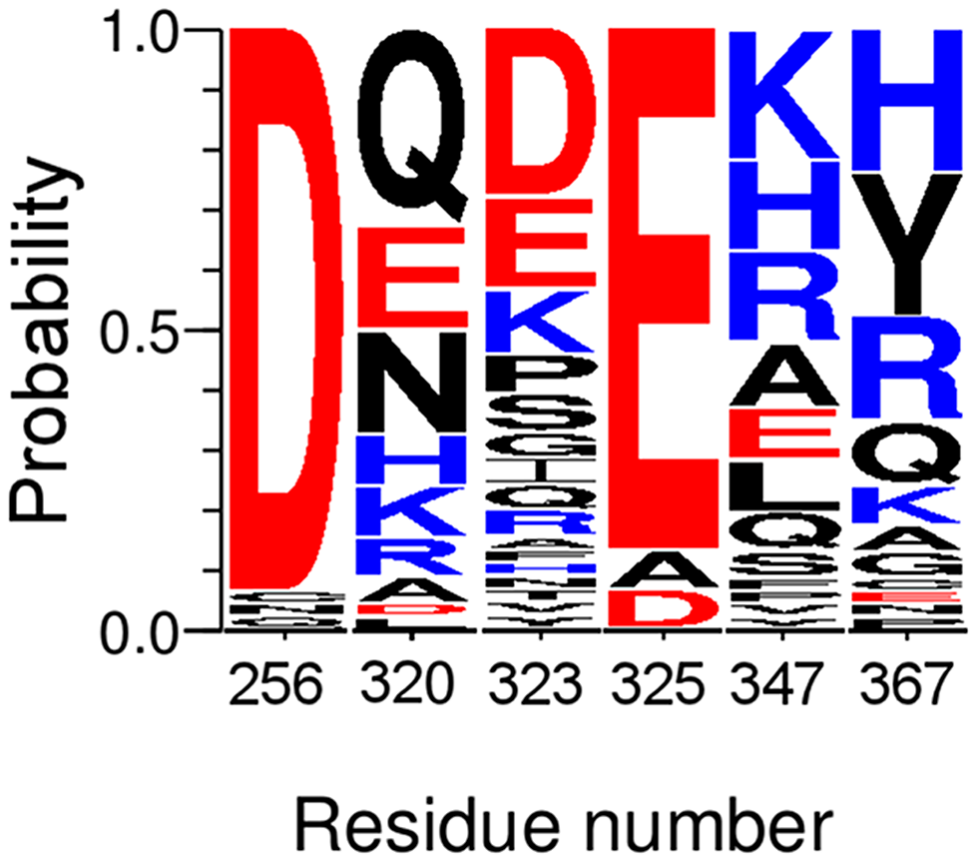
Conservation profile of the residues implicated in identified salt bridges in sequence alignments from heterodimeric nuclear receptors sequences. The alignment is represented using the WebLogo 3 program [70] with the degree of sequence conservation being represented by the height of the amino-acid letter, i.e. the more conserved is the amino acid, the greater is the relative height. Heterodimeric sequences are extracted from the alignment performed by Brelivet et al [29].

Sequence analysis of nuclear receptor LBDs revealed that two sets of differentially conserved residues implicated in salt bridge formation partition the NR superfamily into two classes related to their oligomeric behavior [29]. Following a similar line of reasoning, from the alignment performed in Brelivet et al. [29] we extracted information concerning the amino acids implicated in salt bridge changes observed in the molecular dynamics simulations (see Figure 4). Interestingly, residues implicated in the salt bridge and ion-pair changes upon phosphorylation are mostly conserved in heterodimeric receptors, of which RARα is one. The most conserved residues are D256 and E325 (see Figure 2 and Figure 4) with the percentage 91% and 63%, respectively in heterodimers, and correspond to two important residues in the sequences of nuclear receptors [29]. The other residues D323, R347, E320 and R367 (this latter one conserved as a basic residue R, K, or H) also display an important percentage of conservation in heterodimers with values of 53%, 23%, 41% and 48% respectively.

### Phosphorylation of Ser369 leads to structural changes in helix H9

**Figure 5:**
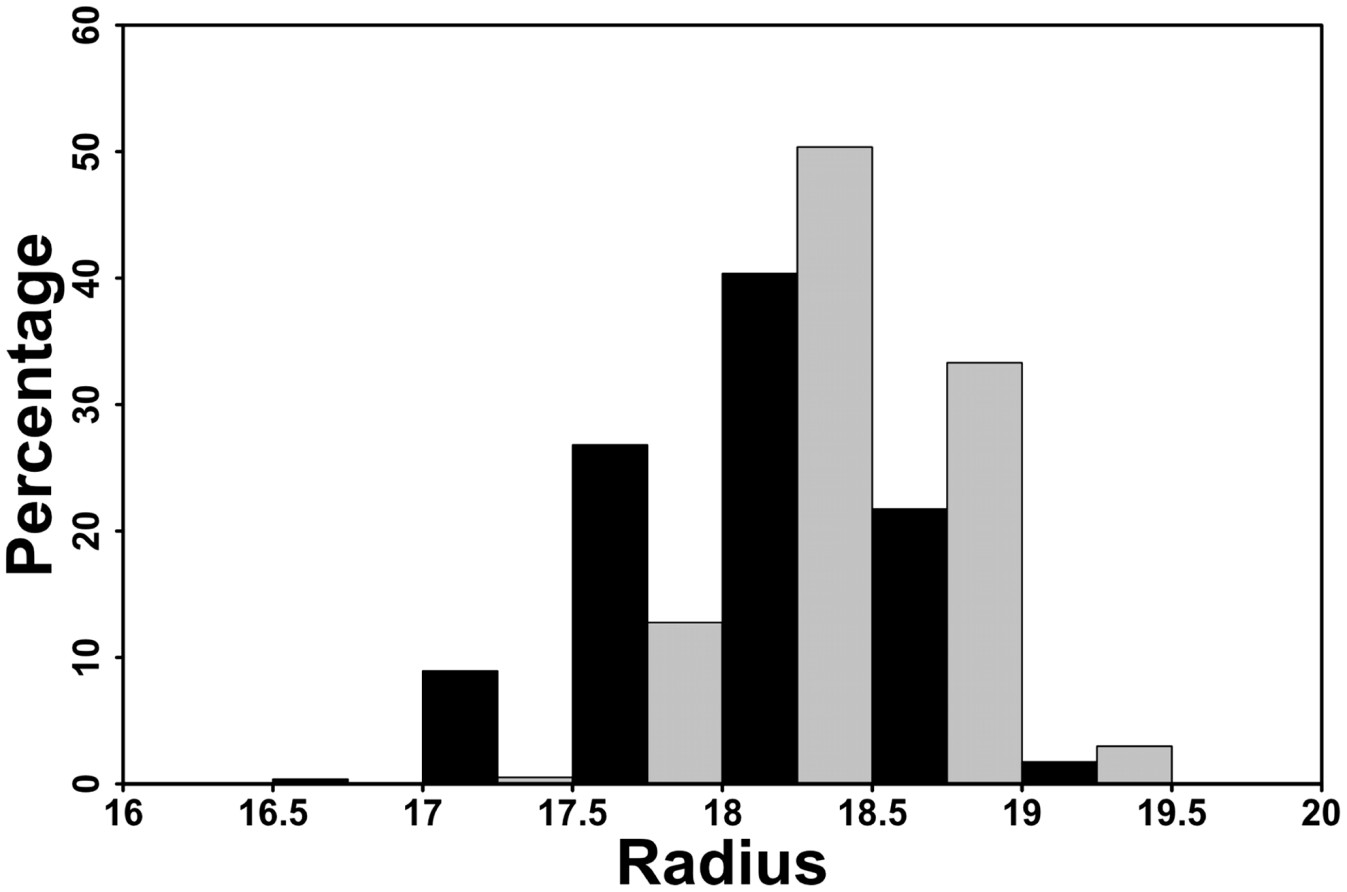
Distribution of the radius of the circle fitted to the α-carbons (Å) of helix H9 in the unphosphorylated (in black) and the phosphorylated (in grey) simulations of RARα. A decrease in the radius of the circle corresponds to an increase in the bend of the helix.

The effect of the S369 phosphorylation on the helix H9 was analyzed by quantifying the helix bend. We estimated the radius of a sphere needed to encircle the Cα atoms of the helix in the non-phosphorylated and phosphorylated forms using the TRAJELIX module [30] in the Simulaid software [31]. A bent helix with a particular sequence requires a smaller sphere than a perfectly extended helix of the same sequence. The average radii of the encircling spheres are 18.20±0.45 Å in the unp-RARα LBD and 18.43±0.34 Å in the p-RARα LBD simulations (see Figure 5). The p-value calculated on these two ensembles using a Student's test is <2.2e^−16^, supporting the subtle differences between the values of the phosphorylated and the non-phosphorylated forms. In Figure 5, we see that 87% of the calculated radii are above 18 Å for p-RARα as opposed to 64% in unp-RARα. This increase in the radius of the encircling sphere fitted to H9 thus indicates the tendency to decrease the bend of the helix, and, by consequence, the extension of helix H9 upon phosphorylation. The individual distributions from the unphosphorylated and phosphorylated simulations are given in Figure S3.

### Phosphorylation of Ser369 alters the angles around helix H9

**Figure 6:**
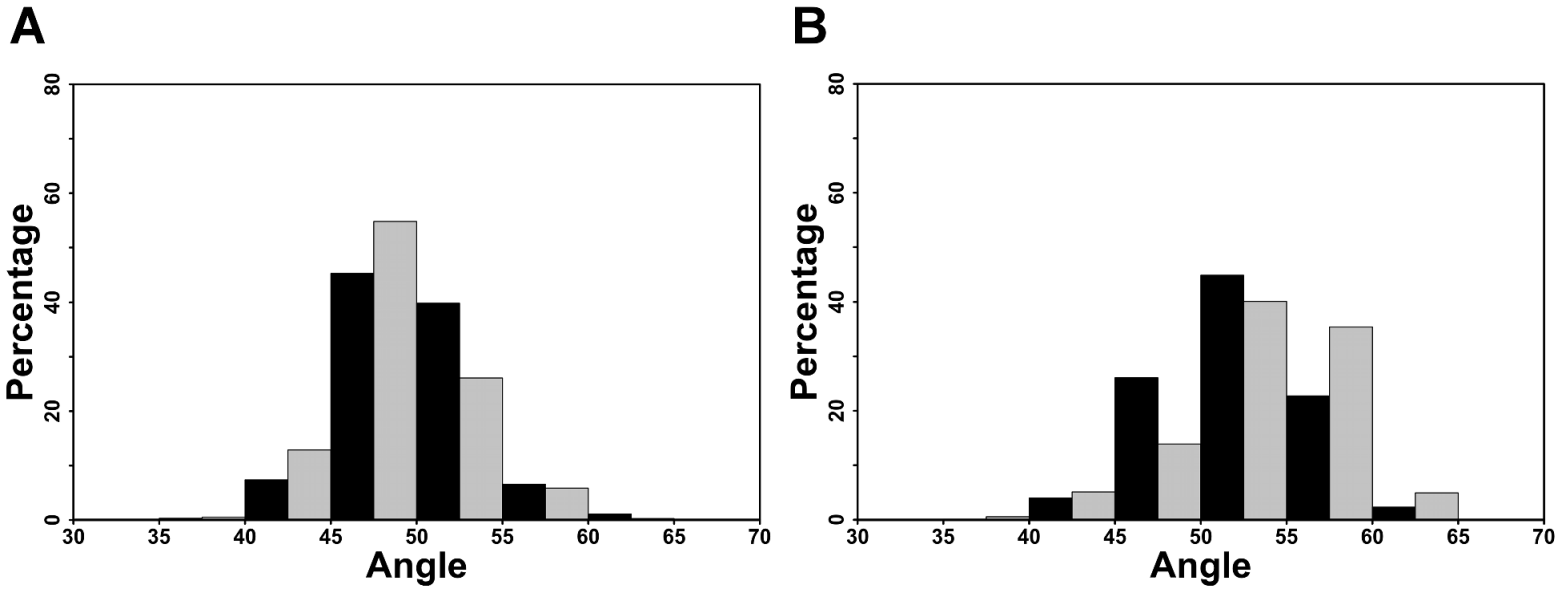
Distribution of the angle values in the unphosphorylated (in black) and the phosphorylated (in grey) simulations of RARα between H9–H10 (A) and H4–H9 (B).

To further investigate the consequences of phosphorylation, we calculated the relative angles formed between helix H9 and helix H10 and between helix H9 and helix H4. The orientations of the helices were calculated using the Chothia-Levitt-Richardson algorithm [32] as implemented in the CHARMM program. Vectors illustrating the angles calculated are presented in Figure S4. Concerning the angle between helices H9 and H10 (see Figure 6.A), we observe a general decrease upon phosphorylation. Indeed, in p-RARα 32% of the conformations had angle values over 50°, while in unp-RARα, 47% of the conformations had angle values above 50°. The average angles between H9–H10 are 48.7±3.7° and 50.0±3.6° for p-RARα and unp-RARα, respectively. The individual angle distributions for the inter-helix angles for H9–H4 and H9–H10 are given in Figures S5.A and B, respectively.

For the angle between H9 and H4 (see Figure 6.B), phosphorylation induced an increase in the angle, with a displacement of the distribution to higher values. Indeed, 80% of the p-RARα conformations have an H4–H9 angle value greater than 50°, as opposed to 70% of unp-RARα conformations. The average values were 53.5±4.5° for the phosphorylated form and 52.2±4.0° for the unphosphorylated one.

We also calculated the values of the angle formed between H8 and H10 (Figure S6). The results show that the average value of the angle between H8 and H10 is 52.1±4.6° in the case of the unphosphorylated receptor and 49.3±3.9° for the phosphorylated form. The percentage of values greater than 50° is 65% for unp-RARα and 40% for p-RARα. This clearly demonstrates that the orientation of H10 with respect to H9 discussed previously is also applicable with respect to H8, supporting the decrease in the angle formed by vectors H8–H10 and H9–H10.

For all angle averages cited above, the Student's t-test yielded p-values less than 2.2e^−16^ so they can be considered to be statistically different. In both cases, one can observe a significant amount of overlap of the distributions, with a slight tendency toward larger (or smaller) values, depending of the angle, as a function of the phosphorylation state. Although small, this shift in population is not inconsistent with the published experimental observations [26], which show that, first of all, un-phosphorylated RARα LBD binds cyclin H, and second, an increase in the binding affinity results from phosphorylation. This is embodied in these figures, which show largely overlapping distributions, but with a slight shift in populations upon phosphorylation.

Overall, the results presented here suggest that the changes in the salt bridge networks involving loop L9–10 lead to a decrease of the angle between helices H9 and H10, which induces a detachment of the loop L8–9 from the helix 4 in the core of the receptor. This, in turn, results in an increase of the angle formed between H9 and H4.

### Phosphorylation alters the structural dynamics of the RARα LBD

To determine if the local changes in the RARα LBD structure described above also affect the structural dynamics and low frequency vibrational modes of the LBD, we analyzed its local and collective structural fluctuations.

**Figure 7:**
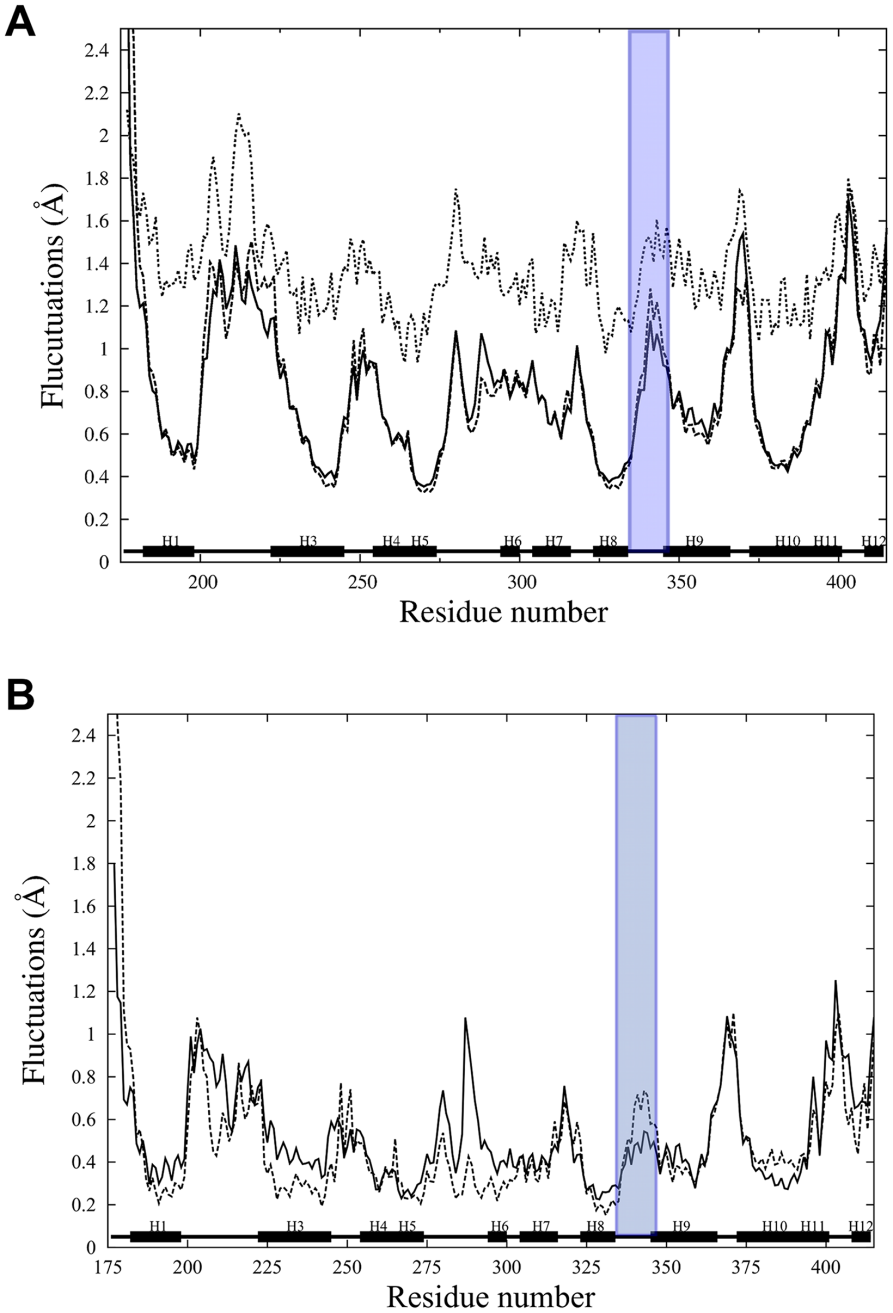
Backbone RMS fluctuations as a function of residue number calculated from the last 40 ns of the molecular dynamics simulations (A) and from the ten lowest frequency modes of the quasi-harmonic analysis (B). Black lines correspond to the average over the three unphosphorylated RARα, dashed lines to the three phosphorylated RARα. In Figure 7.A, dotted lines correspond to the experimental B-factor values. Similar behavior is observed for the fluctuations calculated from simulations and the experimental values. The RMS fluctuations are averaged and displayed by residue. Fluctuations of loop L8–9 are highlighted in purple color.

In order to characterize the local atomic level flexibility, we calculated the by-residue-averaged atomic root mean squared fluctuations (RMSf) from the individual simulations for the backbone atoms. The results show that for the unp-RARα, the RMSf are in general agreement with the trend determined from the experimental B-factors (See Figure 7.A). This analysis further indicates that upon phosphorylation, there is an increase in the fluctuations in the region of the loop L8–9 and the N-terminal part of H9. On the other hand, loop L9–10, where S369 is located, shows a decrease in flexibility upon phosphorylation. This is consistent with the smaller RMSD value for some residues of this loop (see Figure S2). For the purpose of illustration, the RMSf values for residue D341 of loop L8–9 are 1.28 Å and 1.13 Å for p-RARα and unp-RARα, respectively. For residue R370 of loop L9–10, the fluctuations are of 1.2 Å and 1.54 Å for p-RARα and unp-RARα, respectively. Recalling the analysis of structural changes presented above, we found minimal conformational changes upon phosphorylation, however, there is a more measurable difference in the local dynamics, particularly for loop L8–9 and the N-terminal region of H9, as measured by the RMSf. This observation correlates well with the decompaction of the structure in the region of the cyclin binding site discussed above.

Further analysis indicated that this increased flexibility is associated with small, but significant modification of the low frequency motions of the LBD. Quasi-harmonic analyses on the six molecular dynamics of unp- and p-RARα were done in order to characterize the low-frequency motions. The low-frequency modes describe the collective motions of the protein. Averaging over the results from the three simulations of each phosphorylation state, we found the lowest three frequency modes to be 1.13, 1.51, 1.87 cm^−1^ for unp-RARα and 1.07, 1.61 and 1.87 cm^−1^ for p-RARα. Overall, these low frequencies do not differ much between the two forms, which is consistent with our earlier observations that phosphorylation did not alter the dynamics in any major way [26].

From the ten lowest frequency modes of the three phosphorylated and three unphosphorylated simulations, we calculated the RMS fluctuations of the backbone and averaged by residue (Figure 7.B). Interestingly, the fluctuations of loop L8–9 increase measurably when calculated from the low-frequency modes of the p-RARα with respect to the low-frequency modes of unp-RARα. For example, the average fluctuations in the cyclin H docking site reach the value of 0.58 Å in p-RARα in comparison to 0.46 Å in unp-RARα (see Figure 7B). This indicates that phosphorylation affects the low frequency dynamics, which, in turn, modulates the dynamics of the cyclin H binding site.

**Figure 8:**
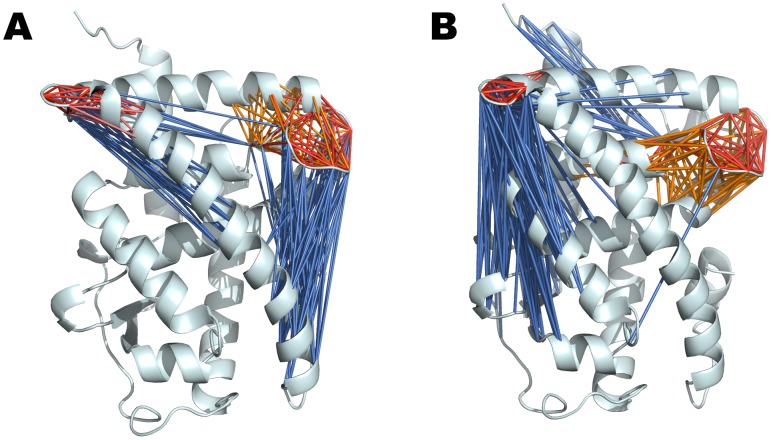
Cross-correlation networks in the unphosphorylated (A) and phosphorylated (B) forms of RARα. The figures show cross-correlations between loops L9–10 and L8–9 and the other structural elements of the LBD by drawing a specific line between two residues if their motion is correlated. The color code corresponds to the value of the cross-correlation coefficient (ccc, see also Figure S5). Marine blue is used for anti-correlated motions (ccc between −0.2 to −0.1), whereas orange (ccc 0.2 to 0.3), salmon (ccc 0.3 to 0.4), light red (ccc 0.4 to 0.7) and red (ccc 0.7 to 1) are used for correlated motions. The changes in the dynamics upon phosphorylation are reflected by the loss of anti-correlated motions connecting loop L8–9 with H11 and the increased anti-correlations of loop L9–10.

To further characterize the structural dynamics of the unp- and p-RARα LBDs, cross-correlation (CC) coefficients were calculated for the six trajectories as described in Material and Methods. These measures range from −1 to 1 and provide information on correlated internal motions of the receptor. In Figure 8, we show a spider web diagram that represents the association of amino acids that are correlated in their motions. The detailed correlation matrix is given in the supplementary material (Figure S7). From this data, one can extract detailed information concerning pathways of correlated motions. What we observe is that upon phosphorylation, the correlation network in the LBD is altered and in particular for loops L8–9 and loop L9–10.

## Discussion

The nuclear receptor RARα is an important player in the regulation of gene transcription and many structure-based studies corroborated the role of ligand binding in the regulatory mechanisms [33]–[36]. More recently, phosphorylation at specific residues also proved to be important and attracted significant attention [37]–[39]. In a non-genomic cascade, RA triggers the activation of the MSK1 kinase, which then phosphorylates S369 located in the LBD. Experimental observations have shown that this phosphorylation leads to an increase in the affinity of the LBD for cyclin H, a component of the cdk7/cyclin H/MAT1 subcomplex of TFIIH. The recruitment of the TFIIH complex through the binding of cyclin H leads to the subsequent phosphorylation by cdk7 of a second serine located in the NTD, and finally the recruitment of the receptor to DNA, a necessary step for gene expression. According to the experimental results [22], [23], phosphorylation increases the binding affinity of cyclin H *in vivo* and *in vitro*. However, no data was available that sheds light on how this phosphorylation structurally activates the cyclin H binding site in RARα. In this context, the present work elucidates details of the allosteric mechanism that is activated upon phosphorylation of S369 of the LBD and results in the enhanced affinity for cyclin H.

In this study we asked the specific questions (i) how phosphorylation of a serine residue in RARα can induce changes in the binding domain of cyclin H, which is located approximately 40 Å away and (ii) what is the mechanism of this allosteric regulation process arising from the post-translational phosphorylation.

Using molecular dynamics simulations, we studied both the structure and dynamics of the RARα LBD. In order to expand the conformational space sampled, we ran multiple molecular dynamics simulations of the phosphorylated and unphosphorylated forms of the LBD, yielding a total of 150 ns for each form of the receptor. From the molecular dynamics simulations, we analyzed the structural and dynamical changes related to phosphorylation in terms of salt bridge and ion-pair formation, helix bending and angles between helices, correlation networks and internal motion.

Phosphorylation is often used as a trigger of allosteric signaling, with signaling mechanisms associated with conformational [40] as well as dynamical changes [41]. In the case of nuclear receptors, experimental data on the estrogen receptor showed very small structural differences between the non-phosphorylated and phosphorylated forms of the LBD (RMSD of 0.59 Å) [42]. X-ray structural analysis of a mutant S/E of RARγ, an RAR subtype highly homologous to RARα, indicates minor structural changes upon introduction of the glutamic acid in loop 9–10 of the LBD, which emulates the phosphorylation (S. Sirigu et al, ms. in preparation). In coherence with the experimental observations, the molecular dynamics simulations of RARα show that no large-scale conformational changes of the LBD occur upon phosphorylation. This suggests that, in the case of the RARα LBD, we have an example of allostery in the absence of significant conformational change.

Allostery can be viewed as a redistribution of an ensemble of pre-existing states upon effector stimulation, with a shift toward a particular state that is favorable, for example, for signal transduction [43], [44]. This view has modulated over the years as a result of the significant improvements in both theoretical and experimental methods and the role of dynamics in allosteric signal transmission is now fully appreciated [45]–[48]. A theoretical background for allosteric signaling in the absence of conformational changes was put forward by Cooper and Dryden in 1984 [49], and experimental examples have since been reported [50], [51].

In the case of the RARα LBD, although no global changes occur, small structural changes near both the phosphorylation site and the cyclin binding site were observed. Phosphorylation modulates the network of salt bridge composed of conserved residues and induces a subtle reorientation of helices H9 and H10 as measured by changes in relative angles. This results in the structural modification of H9 corresponding, in essence, to a straightening of the helix. This straightening of H9 alters the relative position of the N-terminal end of H9 that, along with loop L8–9, makes up the cyclin H binding site. The distance between the N-terminal part of H9 and the C-terminal part of H4 in the phosphorylated RARα is increased, which opens up the region of loop L8–9 and the N-terminal part of H4. These changes were also characterized by the increase in angle formed between H9 and H4.

Taken together, the consequences of the salt-bridge reorganization are small and induce subtle structural changes. Coupling these small population shifts to an allosteric mechanism is not without precedent. Indeed, while the structural changes are small and unlikely to be measurable by standard methods in structural biology, it has been shown in other systems that small changes introduced, for example, by point mutations can lead to significant changes in protein dynamics [52]. In one study, point mutations that were shown to change the specific volume of the protein cyclic AMP receptor protein led to more significant changes in protein compressibility and flexibility as measured by H/D exchange. The changes in specific volume for cAMP were on the order of 0.1%; we estimated changes in RARα from our simulations to be on the same order, around 0.13%. In addition, we also measured an increase in the number of cavities in the average structures of the phosphorylated receptor as measured by the program FPOCKET [53] (data not shown).

These fine structural alterations induced by phosphorylation affects, in turn, the internal dynamics of the receptor by altering the collective motions as shown by the quasi-harmonic analysis of our simulations. The small changes in conformation that occur upon phosphorylation can not be attributed to a specific quasi-harmonic mode, as is often the case for large-scale conformational change, but the change in dynamics of loop L8–9 is captured by changes in the ensemble of low frequency modes. A consequence of these changes, due to phosphorylation of S369, is an increase in the atomic fluctuations of loop L8–9, the cyclin H binding site.

In summary, the scenario proposed here for the allosteric communication pathway is that phosphorylation induces a local ordering of the structure in the region around the phosphorylation site, specifically in loop L9–10, that leads to the release of loop L8–9 via modulation of a the R347-D256 salt bridge, which then permits a greater conformational freedom of L8–9. The observed allosteric communication is subtle in that local modifications in side-chain orientation perturbs the conformational ensemble accessible to the RARα LBD, and the conformational redistribution, although not associated with major structural changes, modifies the intrinsic dynamics of the LBD and favors signal transduction. Evidence exists for other proteins in which allostery occurs not on the backbone level but rather by a rearrangement of side-chains [54], [55]. In the case of RARα, an orchestrated rearrangement of the salt bridges and ion-pairs at either end of H9 was observed, modulating specific structural support and triggering the allosteric communication within the receptor. The changing distribution of ion-pairs upon phosphorylation described in this work illustrates the shift of the conformational ensemble.

Currently, there is no structural information available for the cyclin H/RARα complex and the results presented here are a first step towards the characterization of the interaction between these important binding partners. In the future, we will follow an integrative approach toward the building of an RARα/cyclin H complex in order to better understand how the changes observed here can lead to an enhanced binding affinity. We will characterize the complex and identify important residues for interaction, whose mutations can alter the affinity and thus participate in the non-genomic regulation of the receptor.

## Materials and Methods

### Structure preparation

The X-ray crystal structure of the heterodimeric complex RXR/RAR complexed to all-trans RA and a 13-mer peptide from the Nuclear Receptor Coactivator 2 protein was used (PDBid: 3A9E) [34]. The calculations presented here were limited to the RARα LBD monomer (Figure 1). All heavy atoms were present in the experimental coordinate files. Prior to hydrogen atom placement, the protonation states of all His residues at physiological pH (7.4) were obtained using two different methods of pKa calculation: an empirical method related to the protein structure, PROPKA[56] and the H++ server method [57] based on the Poisson-Boltzmann equation. Both methods yield the same results for protonation states. The final construction of the proton positions, enforcing the protonation states determined in the above continuum dielectric calculations, was done using the HBUILD facility [58] in the CHARMM program [59], [60]. A first energy minimization using a distance-dependent dielectric coefficient and an epsilon of 4, consisted of 100 steps using Steepest Descent method followed by 1000 steps of Adapted Basis Newton-Raphson minimization method, was realized in order to eliminate strong steric contacts before system solvation. The parameters for the phosphorylated form of serine, as well as for the retinoic acid ligand were the same as those previously used [26]. Both forms of RARα (phosphorylated and non-phosphorylated), in complex with the RA ligand and the co-activator peptide, were subject to the same protocol.

### Simulation setup and equilibration

Explicit solvent molecular dynamics simulations of RARα were done using the NAMD program [61] and the all atom force field CHARMM27 [62] with CMAP corrections [63]. After the energy minimization described above, a cubic box of equilibrated TIP3P water molecules with a box length of approximately 75 Å per side was centered on the protein center of mass. Waters overlapping the protein complex were removed. These explicit solvent systems were neutralized by Na^+^/Cl^−^ counterions, additional ion pairs were added to yield a final physiological ionic strength of approximately 0.15 M. Simulations were carried out under periodic boundary conditions and the long-range electrostatic interactions were treated with the Particle Mesh Ewald (PME) algorithm [64]. All bonds between hydrogens and heavy atoms were constrained using the SHAKE algorithm [65], and an atom-based switching function with a cutoff of 12 Å was applied to the van der Waals non-bonded interactions. An integration time step of 1 fs was used for all simulations.

A combined energy mimization-molecular dynamics protocol was used to prepare the solvated system for the molecular dynamics simulations. The water molecules were first relaxed around the fixed protein by 1000 steps of Conjugate Gradient (CG) energy minimization using a constant dielectric coefficient and an epsilon of 1, followed by heating to 600 K over 23 ps, 250 steps of CG, and finally heating over 25 ps to reach the temperature of 300 K. Next, the positional constraints were removed and the entire system was subject to 2000 steps of CG, heating to 300 K over 15 ps. Finally, a production run of 50 ns was simulated, the first 10 ns of the simulations were eliminated from the analysis to ensure a good convergence. This protocol was repeated three times for both the unphosphorylated (unp-RARα) and phosphorylated (p-RARα) form of the nuclear receptor leading to 150 ns total simulation time for each form.

The initial structure used in the simulations of the phosphorylated receptor was that of the WT receptor with a phosphorylated serine in position 369. To further ensure that the simulations of the phosphorylated LBD explore the new local energy basin thoroughly and that no systematic drift of the structure is present during the simulations, we assessed the convergence of the simulations using the ensemble-based approach developed by Lyman and Zuckermann [27]. The procedure can be described as follows: (1) a cut-off distance d_c_ is defined for the calculations and a reference structure S_1_ is picked randomly from the trajectory, (2) S_1_ and all the structures less than d_c_ from it are removed from the trajectory, constituting the set S_1_. These two steps are repeated until there are no conformations left in the trajectory. Next, the trajectory is divided in two groups, in our case according to the time intervals from 10 to 30 ns and from 30 to 50 ns. All the structures in the half-trajectories are clustered according to the set of reference structures, leading to a unique set of structures for a set of given reference structures.

In our case, we clustered each trajectory half using a Cα-RMSD of 1.4 Å as the cutoff distance. This ensured a reasonable total number of reference structures. Lone structures result from the absence of any other structure within the specified cutoff. Here, the criterion used as a measure of good convergence of the simulations was when a low number of lone structures was found in a given trajectory, as was similarly done in earlier work [28]. The convergence calculations were repeated three times for each of the six trajectories using different seeds for the random number generation. For the unphosphorylated and phosphorylated simulations, the number of lone structures found in each set is 2 and 3, respectively. Although this type of convergence evaluation cannot formally rule out the possibility of conformational changes occurring on a timescale longer than that of the simulations, it is a useful test to determine whether a representative ensemble of structures has been generated for both the non-phosphorylated and phosphorylated LDB of RARα with no systematic drifts in the simulations.

### Quasi-harmonic analysis

To characterize the low frequency, collective motions of RARα, as well as changes in these motions as a function of phosphorylation, a quasi-harmonic analysis (QHA) of the molecular dynamics trajectories was performed. The QHA was carried out over the final 40 ns of the simulation using the quasi-harmonic command in the VIBRAN module of the CHARMM program [62]; all the modes were calculated in this analysis (3xN atoms) corresponding to 12,285 modes for the phosphorylated form and 12,276 modes for the unphosphorylated one.

### Analysis of the trajectories

For each simulation, the root-mean-square coordinate difference (RMSD) was calculated, as well as the backbone atomic root mean square fluctuations (RMSf), which were averaged by residue. The RMSf were calculated from the molecular dynamic simulations over the time frames from 10 to 50 ns. These calculated fluctuations were compared to the atomic fluctuations calculated from experimental B-factors.
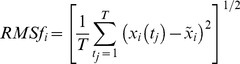
(1)where *t* refers to a specific timeframe, 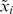 is the reference position of atom *i* (average structure over the time considered), 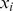 the position of atom *i* at the time *t* and *T* refers to the total number of timeframes used in the calculation of the average, which is related to the time interval for the averaging. Atomic fluctuations were compared to the experimental B factors using:
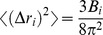
(2)Cross-correlation coefficients *C_ij_* [66] between residues assess the nature of inter-residue motion, that is whether relative motions between the residues *i* and *j* are correlated or anti-correlated. Cross-correlation coefficients can be calculated from normal modes, from quasi-harmonic modes, as well as from molecular dynamic simulations following the equation:
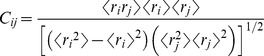
(3)where *r_i_* and *r_j_* are the displacements from the mean position of residues *i* and *j*, respectively. From the *C_ij_* correlation coefficients, which are organized as a matrix, a cross-correlation map was calculated using a color-coded 2D representation. In this representation, *C_ij_* = 1 identifies correlated motions and *C_ij_* = −1 anti-correlated motions. These values give us additional information concerning the global collective motions of RARα.

In this work, we calculated the cross-correlation coefficients directly from the molecular dynamics simulations of 50 ns. Each trajectory was evaluated in blocks of 100, 500 and 1000 ps. For each of these blocks, a mean structure was calculated and the *C_ij_* correlation coefficients were calculated for the backbone atoms. Correlation maps were obtained by averaging the *C_ij_* over all time interval blocks corresponding to the time interval. For the different time intervals, the results were essentially unchanged, so only the results from 500 ps interval are presented here.

From the multitude of LBD crystal structures, it is known that salt bridges play an important structural role. Here, in order to characterize salt bridge formation and stability during the molecular dynamics simulations, we used the “Salt Bridges” plugin for the VMD [67] program, which determines whether a salt bridge is formed. The salt bridge is considered formed if the distance between any oxygen atom of an acidic residue side-chain and the nitrogen atoms of a basic residue side-chain is within a distance of 3.2 Å. The distances were calculated over the last 40 ns of each simulation.

A more detailed structural analysis was done in order to assess any changes to the relative orientation of the secondary structural elements, in particular the helices present in the LBD. The relative orientation of two helices was expressed in terms of an orientation angle as defined in the Chothia-Levitt-Richardson algorithm [32] implemented in the CHARMM program. Axes for helices H4, H9 and H10 were determined based on the respective C_α_ atoms and the cylinder most closely approximating a helix on these atoms was calculated. From this, their relative orientations were determined over the course of the simulations.

A second analysis of helices, which quantified the bend of a helix as the radius of a circle needed to fit the α-carbons [68], was done; the smaller the radius, the larger the helix bend. The software SIMULAID [31], and specifically the TRAJELIX [30] tool, was used to calculate this radius.

Histograms of the distances and the angles obtained are plotted using the R project package [69]. After evaluating the normality of the angles and distances distributions using the Shapiro-Wilk test, we performed a Student's test to assess the statistical difference between the averages of values obtained from the non-phosphorylated and phosphorylated simulations. In supplementary material, we show the distributions of the helix radii from the individual simulations of the un-phosphorylated and phosphorylated LBD (Figure S3). The average radii for the individual simulations are 18.4, 17.8 and 18.4 Å for the unphosphorylated receptor and 18.5, 18.5 and 18.3 Å for the phosphorylated LBD (Figure S3). For reference, the radius calculated using the X-ray structure of helix H9 is 18.5 Å and the value for an ideal alpha-helix of the same sequence built by the PyMol program is 16.6 Å. Thus, in the crystal structure environment, helix H9 of the unphosphorylated receptor is more extended than that of an ideal helix. This is likely due to the alpha-helical sandwich organization of the ligand binding domain of nuclear receptors. One simulation of the unphosphorylated receptor showed a clear shift toward lower values, while the other two show a more subtle shift of the radius toward the ideal value.

## Supporting Information

Figure S1 **Backbone RMSD evolution as a function of time.** Black lines correspond to the average over the three unphosphorylated RARα simulations and grey lines to the three unphosphorylated RARα.
(TIFF)

Figure S2 **RMSD by residue calculated between the initial structure and the average structure calculated over the last 10 ns of the simulations.** Black lines correspond to the average over the three unphosphorylated RARα simulations and dashed lines to the three phosphorylated RARα.
(TIFF)

Figure S3 **Distribution of the radius of the circle fitted to the α-carbons (Å) of helix H9 in the three unphosphorylated (in grey) and the three phosphorylated (in green) simulations of RARα.** A decrease in the radius of the circle corresponds to an increase in the bend of the helix. The averages of the radius for the three unphosphorylated simulations are 18.4, 17.8 and 18.4 Å and for the three phosphorylated simulations are 18.5, 18.5 and 18.3 Å.
(TIFF)

Figure S4 **Illustration of the vectors used in calculating the angle values between H9–H10 and H4–H9 in the simulations.**
(TIFF)

Figure S5 **Distribution of the angle values in the three unphosphorylated (in grey) and the three phosphorylated (in green) simulations of RARα between H4–H9 (A) and between H9–H10 (B).** The averages of the angles between H4 and H9 for the three unphosphorylated simulations are 54.5, 52.7 and 49.3 Å and for the three phosphorylated simulations are 50.2, 54.2 and 56 Å. The averages of the angles between H9 and H10 for the three unphosphorylated simulations are 48, 51.2 and 50.6 Å and for the three phosphorylated simulations are 50.8, 48.4 and 47 Å.
(TIFF)

Figure S6 **Distribution of the angle values in the unphosphorylated (in black) and the phosphorylated (in grey) simulations of RARα between H8–H10.**
(TIFF)

Figure S7 **Cross-correlation maps in the unphosphorylated (upper triangle) and phosphorylated (lower triangle) forms of RARα.** Positive correlated motions range from 0 to 1 (red) and negative ones from −1 (blue) to 0. Time intervals of 100, 500 and 1000 ps were used and averaged over the simulations. Within each state (phosphorylated or not), the results were coherent.
(TIFF)
