# Supplementary figures and images for: Phosphorylation of the Retinoic Acid Receptor Alpha Induces a Mechanical Allosteric Regulation and Changes in Internal Dynamics
Source: PLoS Comput Biol. 2013 Apr 18;9(4):e1003012. doi: 10.1371/journal.pcbi.1003012 (PMC3630199; doi:10.1371/journal.pcbi.1003012)

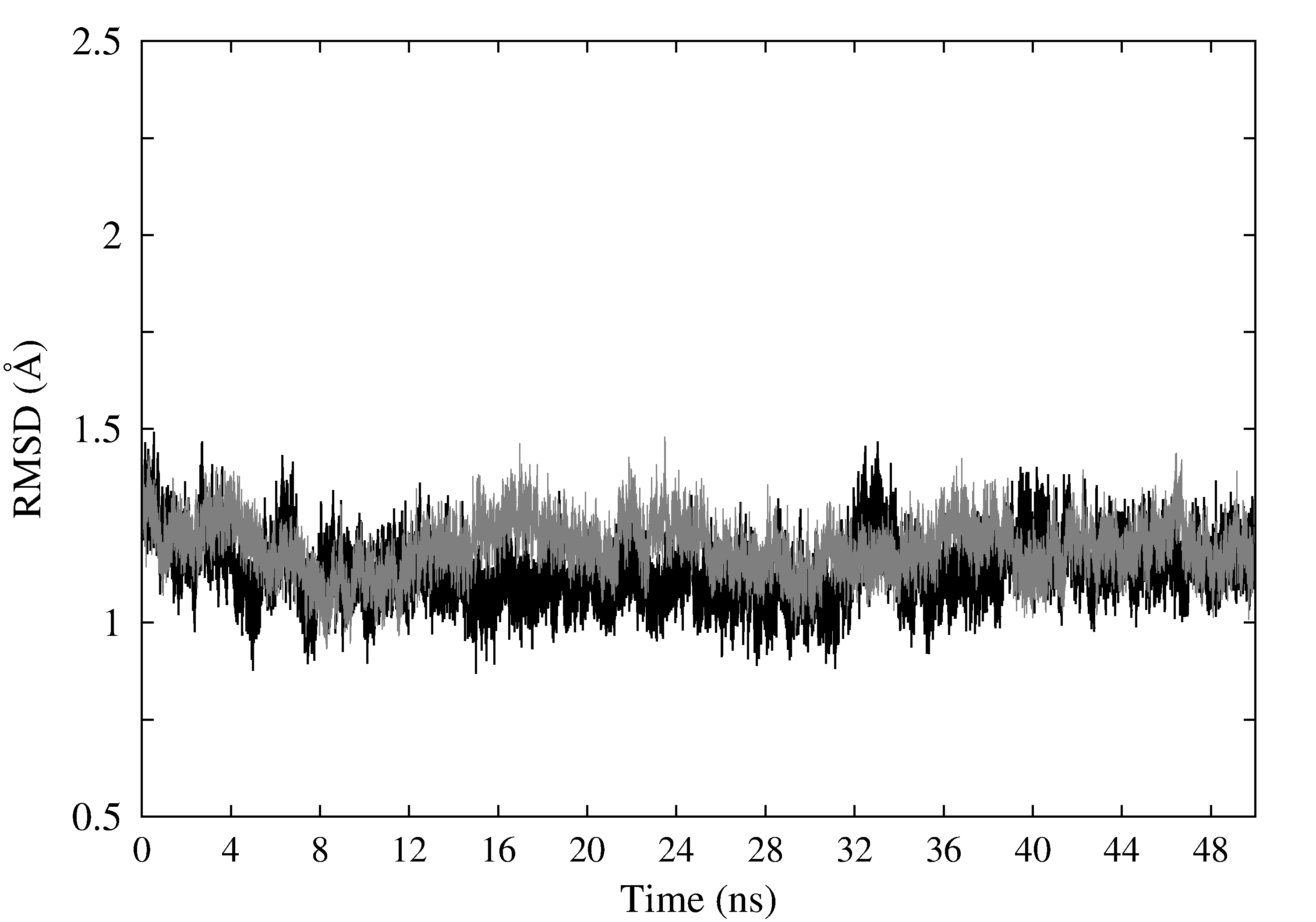

Supplement: Figure S1 — Backbone RMSD evolution as a function of time. Black lines correspond to the average over the three unphosphorylated RARα simulations and grey lines to the three unphosphorylated RARα. (TIFF) [file pcbi.1003012.s001.tiff]

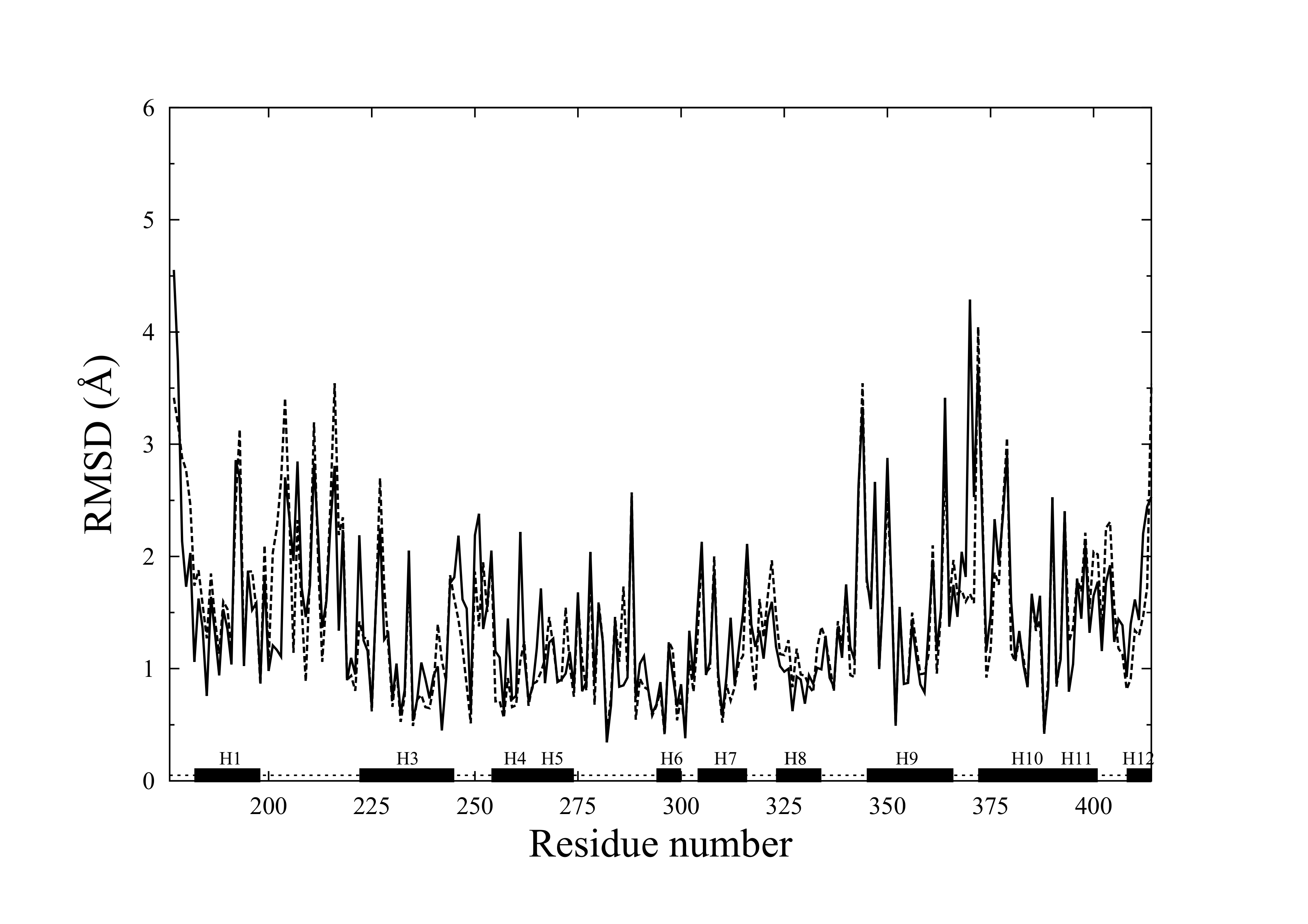

Supplement: Figure S2 — RMSD by residue calculated between the initial structure and the average structure calculated over the last 10 ns of the simulations. Black lines correspond to the average over the three unphosphorylated RARα simulations and dashed lines to the three phosphorylated RARα. (TIFF) [file pcbi.1003012.s002.tiff]

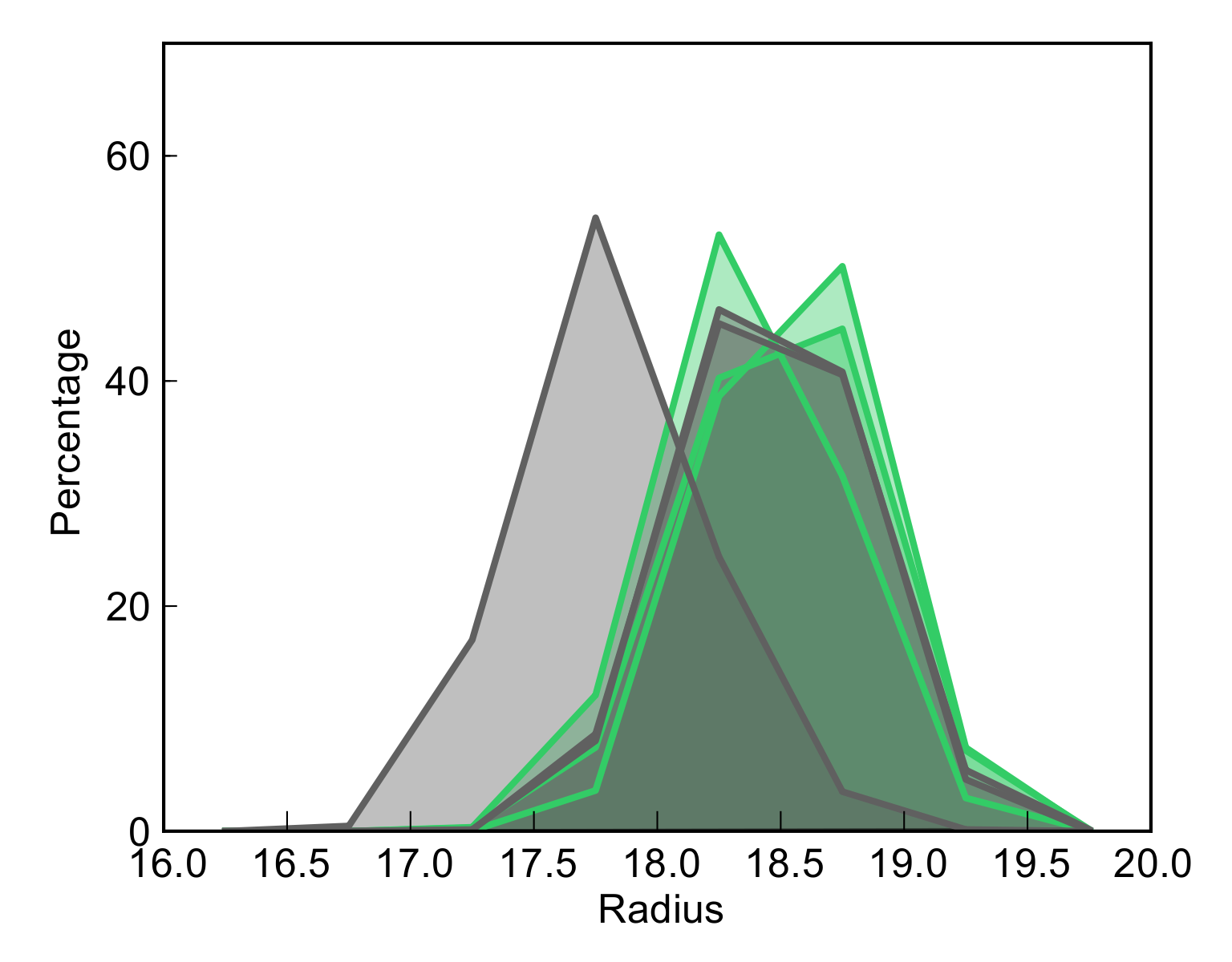

Supplement: Figure S3 — Distribution of the radius of the circle fitted to the α-carbons (Å) of helix H9 in the three unphosphorylated (in grey) and the three phosphorylated (in green) simulations of RARα. A decrease in the radius of the circle corresponds to an increase in the bend of the helix. The averages of the radius for the three unphosphorylated simulations are 18.4, 17.8 and 18.4 Å and for the three phosphorylated simulations are 18.5, 18.5 and 18.3 Å. (TIFF) [file pcbi.1003012.s003.tiff]

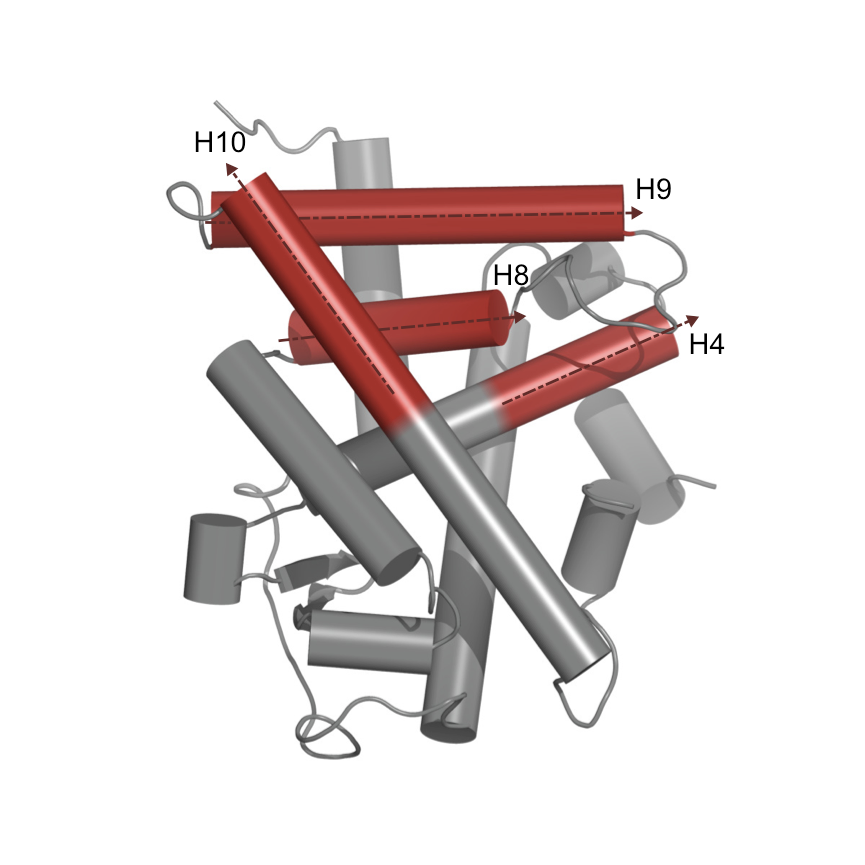

Supplement: Figure S4 — Illustration of the vectors used in calculating the angle values between H9–H10 and H4–H9 in the simulations. (TIFF) [file pcbi.1003012.s004.tiff]

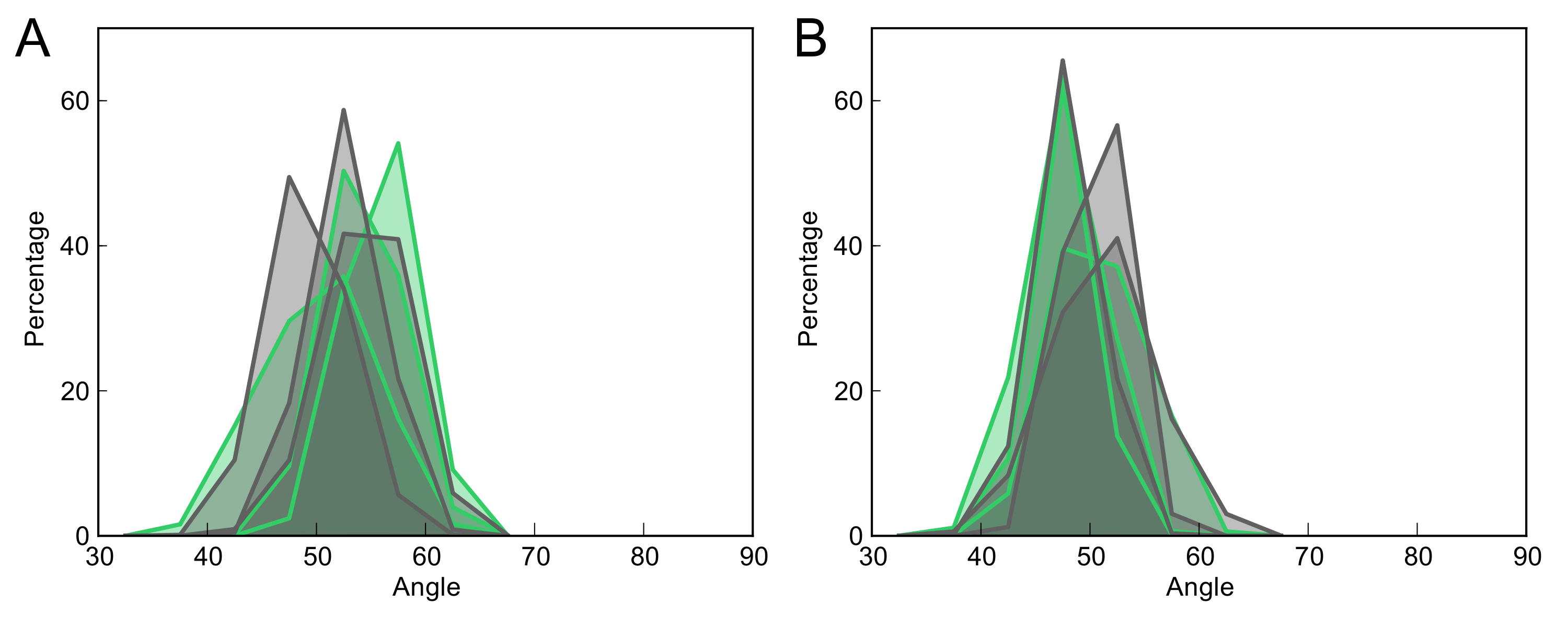

Supplement: Figure S5 — Distribution of the angle values in the three unphosphorylated (in grey) and the three phosphorylated (in green) simulations of RARα between H4–H9 (A) and between H9–H10 (B). The averages of the angles between H4 and H9 for the three unphosphorylated simulations are 54.5, 52.7 and 49.3 Å and for the three phosphorylated simulations are 50.2, 54.2 and 56 Å. The averages of the angles between H9 and H10 for the three unphosphorylated simulations are 48, 51.2 and 50.6 Å and for the three phosphorylated simulations are 50.8, 48.4 and 47 Å. (TIFF) [file pcbi.1003012.s005.tiff]

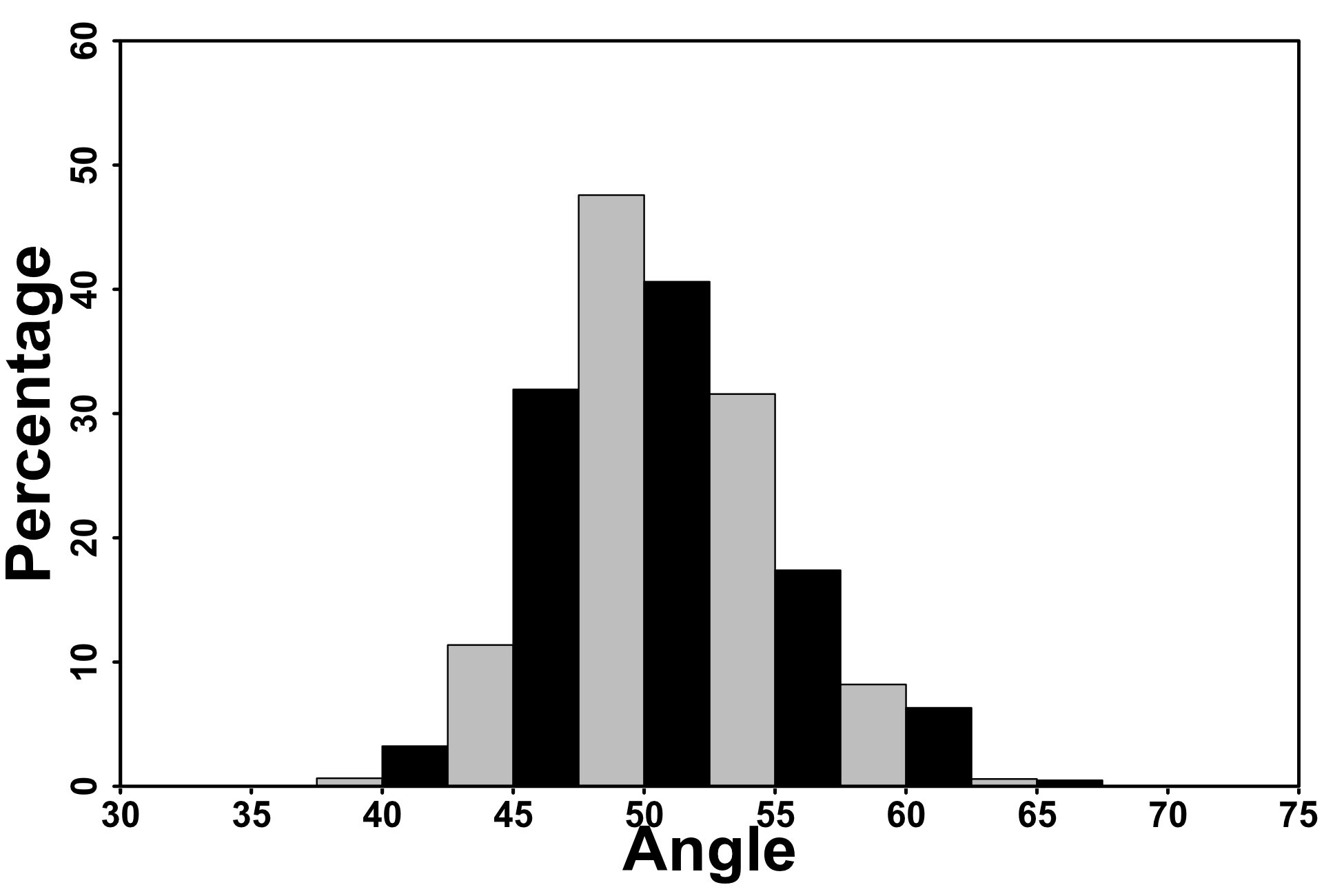

Supplement: Figure S6 — Distribution of the angle values in the unphosphorylated (in black) and the phosphorylated (in grey) simulations of RARα between H8–H10. (TIFF) [file pcbi.1003012.s006.tiff]

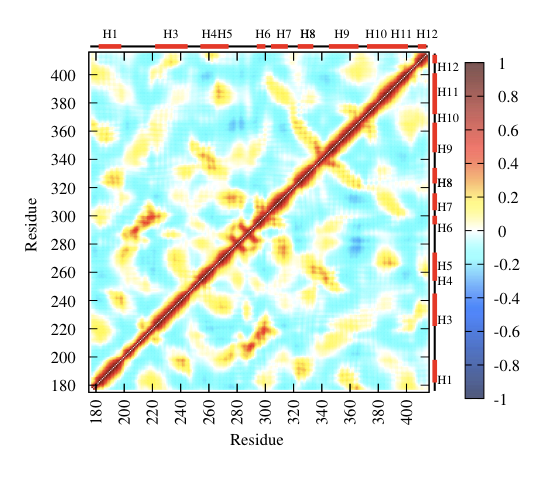

Supplement: Figure S7 — Cross-correlation maps in the unphosphorylated (upper triangle) and phosphorylated (lower triangle) forms of RARα. Positive correlated motions range from 0 to 1 (red) and negative ones from −1 (blue) to 0. Time intervals of 100, 500 and 1000 ps were used and averaged over the simulations. Within each state (phosphorylated or not), the results were coherent. (TIFF) [file pcbi.1003012.s007.tiff]
